# Generation of depolarization-responsive dental stem cell-derived neural-like spheroids using a micropatterned chip platform

**DOI:** 10.1177/20417314261494165

**Published:** 2026-10-07

**Authors:** Normann Misje Roman, Åshild Johansen, Niyaz Al-Sharabi, Inge Fristad, Hiroshi Egusa, Ana Angelova Volponi, Shuntaro Yamada

**Affiliations:** 1 Center of Translational Oral Research, Department of Clinical Dentistry, 1658 University of Bergen, Bergen, Norway; 2 Center for Advanced Stem Cell and Regenerative Research, Tohoku University Graduate School of Dentistry, Miyagi, Japan; 3 Division of Molecular & Regenerative Prosthodontics, Tohoku University Graduate School of Dentistry, Miyagi, Japan; 4 Centre for Craniofacial & Regenerative Biology, Faculty of Dentistry, Oral & Craniofacial Sciences, 4616 King’s College London, London, UK

**Keywords:** Dental stem cells, Micropattern, Organ-on-a-chip, Biofabrication, Regenerative dentistry

## Abstract

Dental stem cells represent an accessible adult stem cell source with neuronal affinity due to their cranial neural crest origin, yet dental-derived neural-like spheroid models often suffer from heterogeneous differentiation, limited organization, and poor reproducibility. Here, we established a stepwise workflow to induce neurogenic differentiation of dental pulp stem cells (DPSCs) and organize them into spatially controlled, depolarization-responsive neural-like spheroids using a micropatterned culture platform. Seven-day neurogenic induction generated Neuro-DPSCs with marked morphological remodeling, neurite-like processes, and increased expression of neural lineage markers including TUJ1, NSE, MAP2, NeuN, and NEFM. In 3D culture, Neuro-DPSCs self-assembled into spheroidal aggregates, while substrate wettability strongly influenced aggregation and outgrowth. Hydrophobic surfaces promoted spheroid formation but limited long-term maintenance due to free-floating aggregation. To improve spatial control, a micropatterned platform was applied to define adhesive contact points, constrain spheroid positioning, and guide inter-spheroid neurite-like network formation. The resulting spheroids showed enrichment of gene networks associated with neurite-like organization, axon guidance, synaptic vesicle biology, neurotransmitter-related pathways, and receptor signaling, together with KCl-induced depolarization-responsive calcium activity. These findings demonstrate that neurogenic priming combined with micropattern-guided assembly provides a controllable workflow for generating spatially organized neural-like DPSC spheroids.

## Graphical abstract

**Graphical Abstract:**
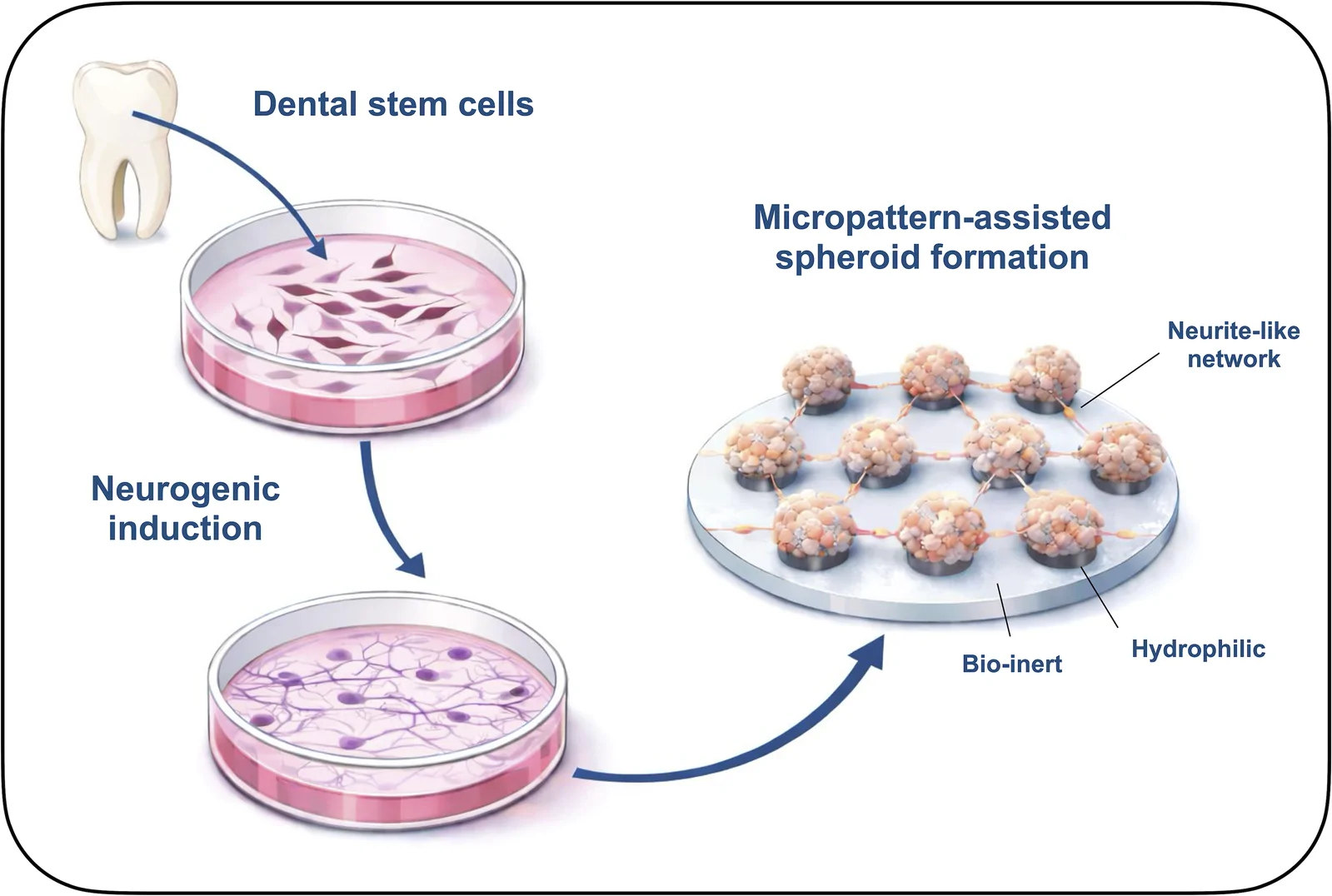

## 1. Introduction

Three-dimensional (3D) culture has become an important bioengineering strategy for investigating how cell–cell interactions, soluble cues, and spatial architecture influence tissue-like phenotypes.^1^ Compared with conventional two-dimensional (2D) monolayers, spheroid-based systems better preserve intercellular contacts and generate local microenvironmental gradients.^2^ In response, 3D neural spheroids have become widely adopted for modelling development, disease, and regeneration, and their integration with microfluidic and organ-on-a-chip technologies is accelerating the move towards more controllable and scalable *in vitro* neural systems.^3,4^ Neural-like spheroids generated from mesenchymal stromal cells (MSC) also provides a complementary, clinically accessible platform for studying early neural-associated phenotypic transitions, cell–cell interactions, and neurotrophic or paracrine responses without requiring primary neurons or pluripotent stem cells.^5,6^ However, many self-organized 3D models still exhibit substantial variability in size, shape, and connectivity, and they often lack deliberate control over how distinct neural microtissues connect to form defined networks.^7^ Approaches that combine 3D self-assembly with spatially defined culture substrates may therefore provide greater control over cellular organization while retaining the biological advantages of spheroid culture.

Dental pulp stem/stromal cells (DPSCs) offer a complementary, clinically accessible adult stem cell source for dental, craniofacial, and neuro de-/re-generative research.^8–10^ DPSCs, a type of MSCs originated from cranial neural crest (CNC), inherit neurogenic competence of CNC cells, including the capacity to adopt neuronal progenitor-like phenotypes and to secrete neurotrophic factors that support neural survival and repair.^8,10^ Multiple studies have reported neural induction protocols for DPSCs, such as the use of serum free neural media, small molecules, and growth factor-driven approaches, as well as sphere formation assays that enrich for neural progenitor-like subpopulations.^11,12^ These observations, together with encouraging preclinical studies in peripheral nerve and central nervous system injury models, position DPSCs as a promising cell source for neural repair and for building human relevant neural models.^9^ However, despite the advancement, DPSC neurogenic phenotypes are still frequently inferred from a limited set of immunophenotypic markers and morphological changes, which may overlook the overall maturation state and lineage fidelity of the induced cells.^13,14^ Moreover, reported neural induction strategies for DPSCs vary widely in medium composition, induction timing, and culture format, and donor- and subpopulation-level heterogeneity further contributes to variable outcomes, making robust cross-study benchmarking and reproducibility challenging.^11^ In this context, comprehensive phenotyping that integrates high-content marker profiling with molecular signatures of neural programs and quantifiable, engineered network formation can strengthen the interpretability and translatability of DPSC-derived neural models.

Engineering strategies that utilize 3D culture with microfabrication offer a route to reduce this variability and to impose designed architecture on developing organoids. Micro- and nanofabricated substrates can control cell adhesion, morphology, and cytoskeletal organisation, which in turn regulate lineage specification and network assembly.^15^ Patterned substrates and microcontact printing have been used to guide neurite outgrowth, constrain neuronal polarity, and create reproducible circuits, including the organisation of synaptogenic adhesion molecules in defined geometries.^16,17^ However, most micropattern based neural network platforms have been optimised for primary neurons or pluripotent stem cell-derived neurons in 2D, whereas translation to adult stem cell sources and to 3D spheroid-based assemblies remains less explored. In parallel, DPSC-derived spheroids are typically generated by round-bottom low-attachment aggregation or hydrogel embedding, which yields stochastic spheroid placement and largely uncontrolled size, shape and inter spheroid connectivity.^18–20^ As a result, there is a practical gap between (i) the biological promise of DPSCs as CNC-related, patient-relevant cells and (ii) the availability of reproducible, geometry defined, and network competent 3D platforms that can enable interconnected DPSC-derived neural-like spheroids and organoids.

In this study, we designed the micropatterned chip-assisted approach for the generation of neural-like DPSC spheroids that can form interconnected neurite networks in a self-assembled yet geometry-controlled manner. We describe a stepwise biofabrication with thorough characterisation of DPSCs after neural induction and neural-like spheroid formation, including high-throughput marker profiling of key pathways linked to neural progenitor regulation, axon guidance, synaptic transmission, neuronal migration and polarity, and functional assessment. This study fills a current knowledge gap by proposing a reproducible and scalable strategy to build networked neural-like DPSC spheroids whose spatial organisation is designed rather than incidental and revealed in-depth characterization of developed neural-like DPSC spheroids.

## 2. Materials & Methods

### 2.1. DPSC isolation, expansion, and MSC characterization

Ethical approval for the use of human material was obtained from the Regional Committee for Medical and Health Research Ethics in Norway (REK vest; 2009/610). Dental pulp tissue was harvested from premolars of juvenile donors following informed consent. All teeth had closed apices, were caries free, and showed no evidence of inflammation or pathology in the pulp or periodontal tissues. The pulp tissue was enzymatically digested with collagenase type I (4 mg/mL) and dispase (2 mg/mL). Negative result for mycoplasma was confirmed by Hoechst 33258 staining prior to the study. DPSCs were maintained in Dulbecco’s Modified Eagle’s Medium (DMEM; 10566016, Gibco, USA) supplemented with 10% fetal bovine serum (FBS; 10270-106, Gibco, USA), 1% penicillin/streptomycin (SV30010, HyClone, USA), and 0.1 mg/ml Normocin (ant-nr-05, InvivoGen, USA) at 37°C in 5% CO_2_ under humidified conditions. For the study unless otherwise mentioned, DPSCs from five female donors were pooled in equal cell-number proportions at passage 2 to average donor-to-donor variability. Experiments were performed using passage 3–5 DPSCs.

MSC characterization was conducted in accordance with ISCT (International Society for Cell & Gene Therapy) MSC criteria for all the donors independently before pooling as well as post-pooling at passage 3^21^ (Appendix Figure S1). Briefly, their MSC phenotype was identified by testing 3 negative (CD34, CD45, and HLA-DR) and positive (CD73, CD90, and CD105) markers each by flow cytometry. Their multilineage differentiation was induced for osteogenesis, chondrogenesis and adipogenesis as previously demonstrated.^22^

### 2.2. Neurogenic induction of DPSCs in monolayer culture

The experimental overview is displayed in Figure 1A. DPSCs were seeded onto 6 well plates coated with laminin (354404, Corning, USA) at the density of 5000 cells/cm^2^ for neurogenic differentiation. When the cells reached 80% confluency, the growth medium was replaced with serum-free MSC Neurogenic Differentiation Medium with proprietary supplements (C-28015, PromoCell, USA), which were tested previously to induce neural-like phenotypes of dental stem cells in monolayered culture systems and characterized.^23–25^ The neurogenic induction lasted for 7 days.

**Figure 1.**
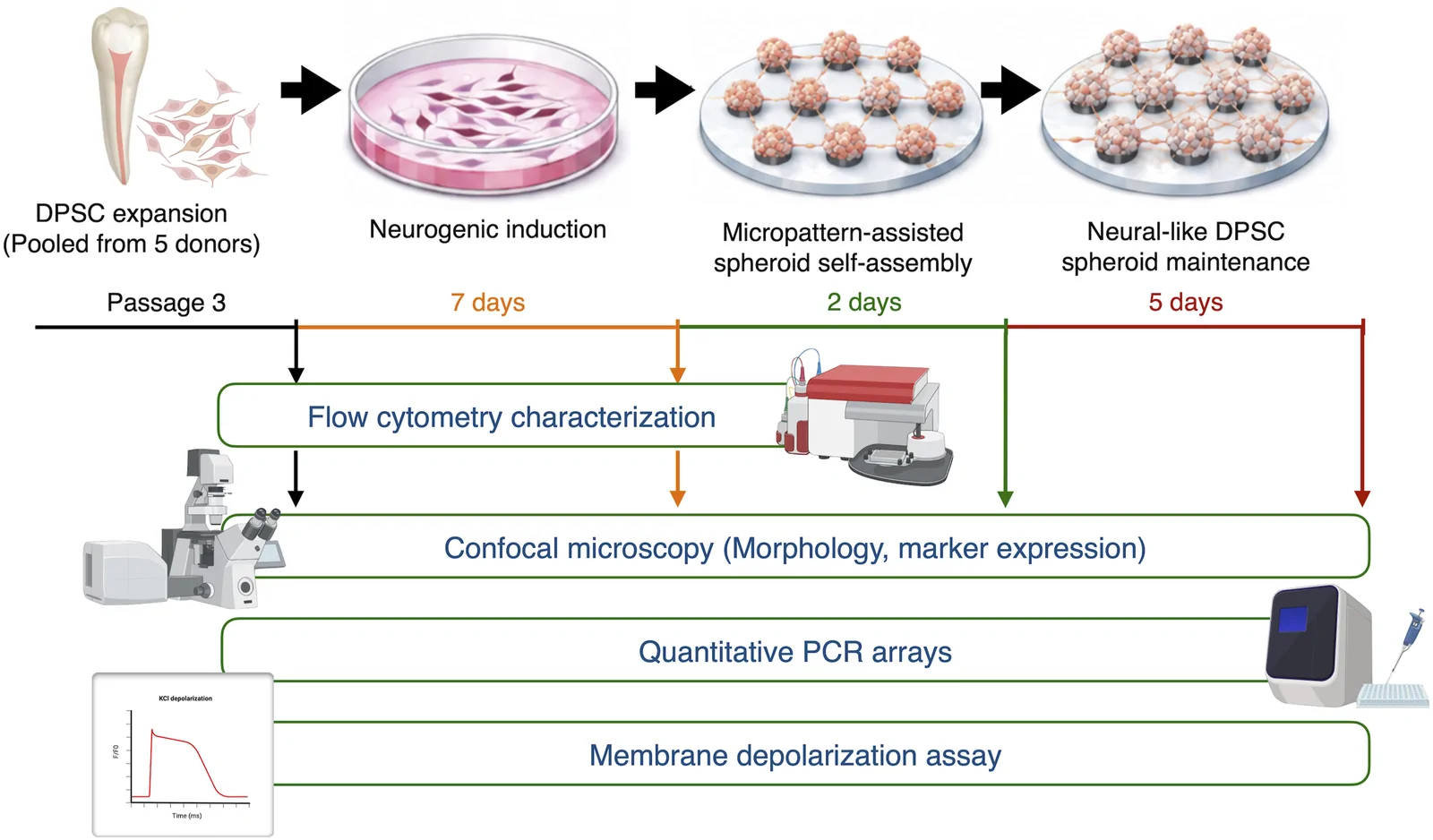
Schematic illustration of the study design and analysis workflow.

### 2.3. Generation and maintenance of neural-like DPSC spheroids

After neurogenic induction, neuro-induced DPSCs (Neuro-DPSCs) were trypsinized, resuspended in the induction medium, and seeded for subsequent spheroid formation on the following three substrates for unsupervised and supervised (i.e. micropattern-assisted) formation.

For unsupervised formation, neuro-DPSCs were seeded at 1 ×10^6^ cells/ml, 300 μl in total on polymeric coverslip with and without tissue culture treatment (80806, 80801, ibidi GmBH, Germany), resulting in hydrophilic and hydrophobic surfaces.

For micropattern-assisted spheroid formation, bioinert polymeric coverslip was micropatterned (200 μm circles, 600 μm pitch, hexagonal; available as 83813, ibidi GmBH, Germany) to allow organized spheroid arrangement. Neuro-DPSCs at 1 ×10^6^ cells/ml, 300 μl in total, were seeded per well and maintained in the neurogenic induction medium for 2 days to stabilize the spheroid. Subsequently, the neuronal spheroids were either continuously maintained in neurogenic induction medium (NGM; C-28015, PromoCell, USA) or previously optimized serum-free neurogenic maintenance medium (NMM). NMM consisted of Neurobasal medium (21103049, Thermo Fisher, USA) supplemented with 1× B27 (175044, Thermo Fisher, USA), 1× GlutaMAX (35050061, Thermo Fisher, USA) 50 ng/ml human recombinant beta-NGF (HY-P7660, MedChemExpress, USA), 20 ng/ml recombinant human BDNF (HY-P7116A, MedChemExpress, USA), recombinant human Neurotrophin-3 (HY-P70456, MedChemExpress, USA), and 100 µM L-ascorbic acid (A8960, Sigma-Aldrich, USA).

### 2.4. Flow cytometry

For MSC marker characterization, DPSCs were typsinized and resuspended in a blocking buffer consisting of 10% Normal Goat Serum (NGS) in PBS on ice. For intracellular markers for neuronal lineages, the cells in suspension were fixed in 2% paraformaldehyde (PFA) for 15 minutes at room temperature followed by permeabilization and blocking in 10% NGS and 0.1% Triton-X100 in PBS for 30 minutes on ice.

A total of 2×10^5^ cells in 100 μl staining buffer were then incubated with primary antibodies for 45 minutes on ice. With unconjugated antibodies, the cells were washed twice and subsequently incubated with secondary antibodies for 45 minus on ice. The list of antibodies and concentrations used in the study is provided in Table S1. Fluorescence was measured on Accuri C6 flow cytometer (BD Biosciences, USA).

### 2.5. Reverse transcription-quantitative polymerase chain reaction (RT-qPCR) and data analysis

To evaluate marker expression of DPSCs, neuro-DPSCs, and NGM and NMM-maintained spheroids, we designed a custom RT-qPCR array using the TaqMan platform (4413257, Applied Biosystems, USA). The full gene list and corresponding assay IDs are provided in Table S2. RNA extraction, cDNA synthesis, and qPCR cycles were performed as described previously.^26^ Total RNA was collected from three independent experiments for each group, with each treated as one biological replicate (n = 3).

Gene expression was analyzed using the ΔΔCt method.^27^ Briefly, Ct values for each target gene were first normalized to the average of endogenous controls (GAPDH, HPRT1, IPO8, TBP, TFRC, GUSB) to obtain ΔCt. ΔΔCt values were then calculated relative to the control group, and fold changes (FC) in expression were reported as 2^(-ΔΔCt).

### 2.6. Calcium imaging

Cell excitability was assessed using a Fluo-4 Calcium Imaging Kit (F10489, Thermo Fisher Scientific, USA) in accordance with the manufacturer’s protocol. Cells were incubated with Fluo-4 AM in Hank’s balanced salt solution at 37°C for 30 min and then maintained at room temperature for a further 30 min to permit dye de-esterification. Probenecid was added to reduce cytosolic dye extrusion. After washing, baseline fluorescence was recorded, and cells were then stimulated with 50 mM KCl to induce membrane depolarization and calcium influx. Time-lapse fluorescence imaging was performed using a Nikon Eclipse Ti microscope equipped with a 10× objective, and the acquired images were analyzed in FIJI/ImageJ. For quantitative analysis, 50 individual cells per group were evaluated in 2D cultures, while 10 spheroids per group were analyzed in 3D cultures.

### 2.7. Real-time spheroid formation monitoring

Spheroid forming ability of DPSCs and neuro-DPSCs on the hydrophobic substrate was analyzed using a real-time imaging system (Incucyte S3, Sartorius, Germany) to evaluate the size growth of neuronal spheroids over the period of 36 hours post seeding. The quantification was conducted on Incucyte Spheroid Analysis Software Module using all the detected particles/spheroids in 24 ROIs per group in total.

### 2.8. Immunofluorescence and confocal microscopy

Samples were fixed in 4% PFA for 15 minutes followed by permeabilization in 0.1% Triton X-100 for 15 mins and blocking in 10% NGS for 45 minutes at room temperature. The samples were incubated in a staining solution consisting of primary antibodies in 0.05% Triton X-100 and 5% NGS in PBS overnight at 4 degrees in dark. The antibodies used were AlexaFluor 488 anti-TUBB3 (TUJ1) antibody (1:250; 53-4510-82, Thermo Fisher, USA), anti-NEFM antibody (1:250; MA5-32613, ThermoFisher, USA), anti-GAD1 antibody (1:200; CL594-67648, Proteintech, USA), and anti-Synaptophysin (SYP) antibody (1:200; CL647-17785). Where applicable, the samples were then incubated with secondary antibody (AlexaFluor 647 anti-rabbit antibody; 1:500; A32773, Thermo Fisher, USA) overnight at 4 degrees in dark. The nuclei and actin cytoskeleton were counterstained with Hoechst 33342 (B2261, Merck, Germany) and ATTO565 Phalloidin (94072, Sigma-Aldrich, USA), respectively. For fat droplet staining, Bodipy 493/503 (D3922, Thermostat, USA) was used.

Two-dimensional samples and three-dimensional spheroids were imaged as z-stacks using a Leica SP8 confocal microscope and a Leica Stellaris confocal microscope (Germany), respectively, both equipped with 10× and 40× objectives. For wide-field acquisition, tile scanning was performed with 15% overlap. Post-acquisition processing and morphometric analysis were carried out in FIJI/ImageJ.^28^

### 2.9. Statistical analysis

All qualitative and quantitative experiments were performed using three independent biological replicates unless otherwise stated. Statistical analyses were performed on GraphPad Prism 10. For RT-qPCR assays and arrays, two-way analysis of variance (ANOVA) followed by Šídák’s multiple-comparisons test was used. For Incucyte time-course measurements, two-way ANOVA was used with Šídák’s multiple comparisons test. For comparisons between two independent groups, a two-tailed Welch’s t-test was performed. Data are presented as the mean ± standard deviation (SD) unless otherwise stated. A p value < 0.05 was considered statistically significant. Numerical data including sample size and original values used for statistical tests are available in Appendix Table. S3.

## 3. Results

### 3.1. Neurogenic induction generates neuro-DPSCs with neural-like morphology and marker expression

DPSCs were subjected to a 7-day neurogenic induction to generate neuro-DPSCs (Figure 2A). Compared with control DPSCs, neuro-DPSCs exhibited rapid and pronounced morphological remodeling (Figure 2B, B′). By day 3, the cells showed reduced cytoplasmic spread and developed elongated, neurite-like processes. By day 7, these features became more evident, with small soma-like cell bodies and long, branching projections that formed interconnected networks. Immunofluorescence staining further supported cytoskeletal and neuronal marker reorganization after induction. While control DPSCs displayed predominantly stress fiber-like F-actin organization with limited neuronal marker expression, neuro-DPSCs demonstrated extensive process formation and upregulation of βIII-tubulin (TUJ1) and NEFM along the elongated extensions (Figure 2C). Marker profiling demonstrated, while control DPSCs robustly expressed neural-associated and mature neuron markers including Nestin, Tuj1, MAP2, NeuN, and NEFM at the baseline, neurogenic induction further increased the expression level. On the contrary, glial/immune markers (e.g., IBA1 and GFAP) remained low or were detected only in a minor subpopulation in both DPSCs and neuro-DPSCs (Figure 2D, E).

**Figure 2.**
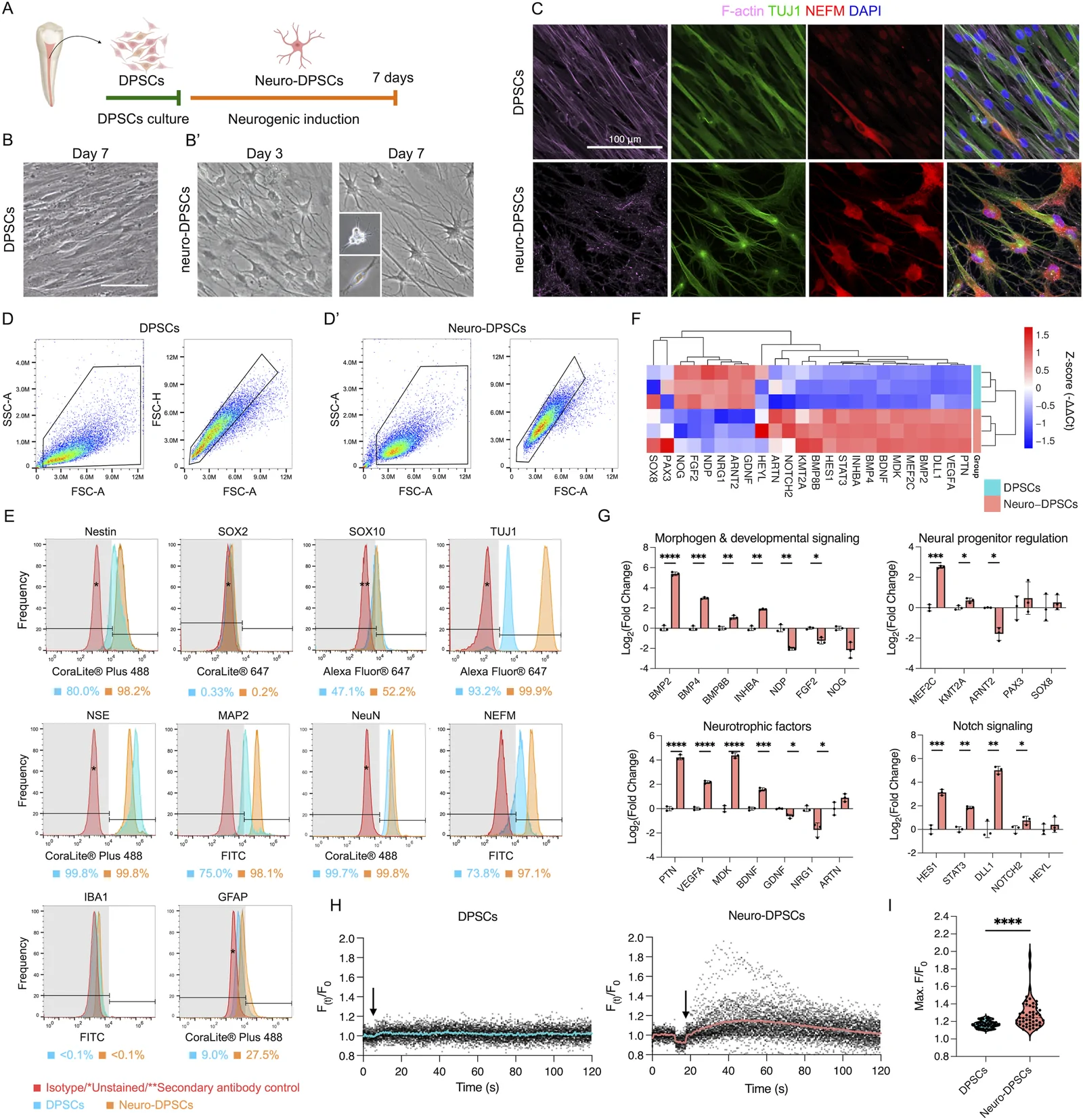
Neurogenic induction generates neuro-DPSCs with neuronal-like morphology and upregulation of neural gene expression. (A) Schematic of the experimental workflow. (B, B’) Representative phase-contrast image of control DPSCs and neuro-DPSCs. (C) Immunofluorescence images of DPSCs and neuro-DPSCs. (D, D′) Flow cytometry gating strategy for DPSCs and neuro-DPSCs showing singlet gating. (E) Marker profiling of DPSCs and neuro-DPSCs for neuronal progenitor (Nestin, SOX2, SOX10), pan-neuronal cytoskeleton (TUJ1), intermediate to mature neuron (NSE, MAP2, NeuN, NEFM), microglia (IBA1) and glia (GFAP). (F) Heatmap of RT-qPCR results showing differential expression of neurodevelopmental and neurogenic genes in DPSCs versus neuro-DPSCs with unsupervised hierarchical clustering (Euclidean distance). (G) mRNA expression of selected gene groups. Statistical analysis was performed by two-way ANOVA with Šídák’s multiple-comparisons test (n = 3). (H) Fluo-4-based calcium imaging traces showing changes in intracellular calcium levels in response to KCl stimulation (arrow). Each dot represents single cell (n = 50) while the lines indicate average values (I) Violin plot of maximum F/F_0_ values. Statistical analysis was performed by Welch’s t-test (n = 50). *p < 0.05, **p < 0.01, ***p < 0.001, ****p < 0.0001.

Consistent with these phenotypic changes, RT-qPCR analysis revealed a distinct transcriptional profile separating non-induced DPSCs and neuro-DPSCs (Figure 2F). Neuro-DPSCs showed a trend of the upregulation of morphogen and developmental signaling genes, neural progenitor regulation-related genes, and neurotrophic factors, alongside modulation of Notch pathway-associated genes (Figure 2G), indicating activation of neurodevelopmental programs during the induction process. Calcium imaging demonstrated that a population of neuro-DPSCs were more responsive to KCl stimulation than control DPSCs (Figure 2H). While control DPSCs showed little-to-modest excitability, neuro-DPSCs displayed a noticeable transient increase in F/F_0_ after stimulation, followed by a gradual decrease. Consistently, the maximal F/F_0_ value was significantly higher in neuro-DPSCs than in control DPSCs (p < 0.0001), suggesting acquisition of enhanced depolarization-responsive calcium dynamics following neural induction on monolayer culture (Figure 2I).

### 3.2. Neuro-DPSCs robustly form neural-like spheroids and thicker three-dimensional assemblies

To generate 3D neural-like spheroids, control DPSCs and neuro-DPSCs were detached and then re-seeded onto hydrophilic and hydrophobic surfaces (Figure 3A). Time-lapse quantification demonstrated that neuro-DPSCs rapidly self-organized themselves into larger spheroidal aggregates on the bioinert surfaces while control DPSCs adhered on the substrate and did not form a spheroid (Figure 3B, C). Noteworthily, the self-organized spheroid in the wells demonstrated significant variation in size as indicated by a large standard deviation.

**Figure 3.**
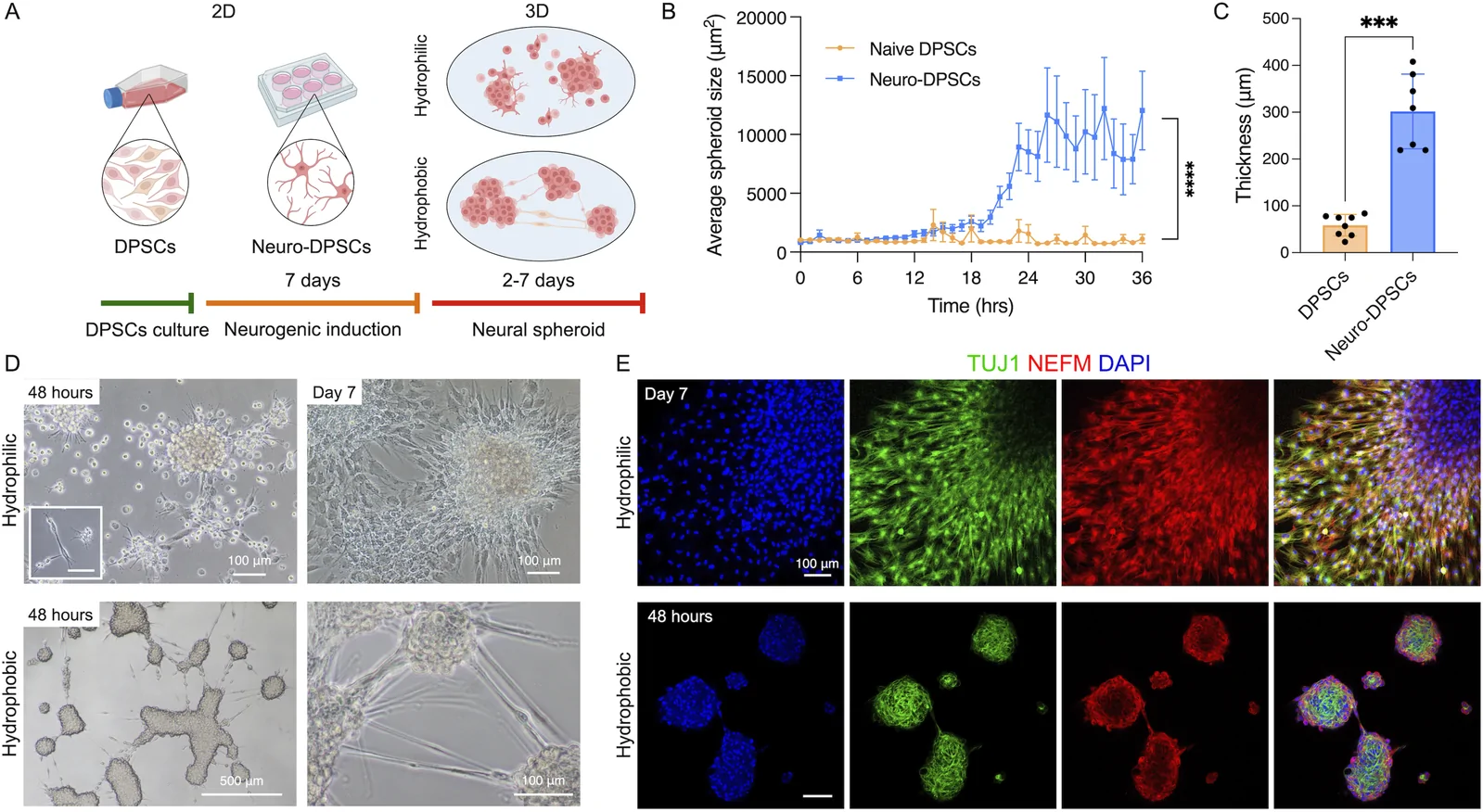
Neuro-DPSCs form neural spheroids and thicker 3D assemblies in a wettability-dependent manner. (A) Experimental workflow for 3D spheroid generation. (B) Time-lapse quantification of average spheroid size analyzed by IncuCyte Spheroid Analysis Module, showing rapid self-assembly of neuro-DPSCs into neural-like spheroids while naïve DPSCs lacks self-assembly capacity. For statistics, two-way ANOVA was used with Šídák’s multiple comparisons. (n=8 for DPSCs, n=7 for Neuro-DPSCs). (C) Quantification of spheroid thickness measured from confocal z-stack images. Statistical analysis was performed by Welch’s t-test (n = 6). (D) Representative phase-contrast images illustrating the effect of substrate characteristics on aggregation and outgrowth. (E) Representative immunofluorescence images of 3D cultures stained for TUJ1, NEFM, and nuclei. ***p < 0.001, ****p < 0.0001.

Substrate wettability strongly influenced spheroid organization and subsequent outgrowth. On hydrophilic surfaces, neuro-DPSCs formed multiple aggregates/colonies at 48 h, followed by spreading and dense cellular outgrowth by day 7 (Figure 3D). In contrast, on hydrophobic surfaces, cells preferentially assembled into more compact and discrete spheroids, with pronounced neurite-like network structures connecting the spheroids. However, because the spheroids remained non-adherent, fluid movement during medium exchange, particularly that generated by pipetting, rapidly disrupted the spatial organization of these assemblies (Appendix Figure S2). Immunofluorescence analysis confirmed neuronal marker expression within these 3D cultures. At day 7 under hydrophilic conditions, extensive cellular outgrowth showed strong staining for TUJ1 and NEFM (Figure 3E). Under hydrophobic conditions, compact spheroids at 48 h also displayed clear TUJ1 and NEFM positivity throughout the aggregates, whereas culturing such structures over 48 h was not achievable due to the fact the spheroids remained free-floating without adhesion to the substrate. It is also noteworthy that due to staining procedure, spheroid network and overall morphologies on the hydrophobic surfaces were largely abolished.

### 3.3. Micropatterned culture enables organized neural-like spheroid assembly and maintenance

To overcome the limited long-term culture of free-floating spheroids on hydrophobic surfaces, neuro-DPSCs were seeded onto a micropatterned chip with the “points of adhesion” to spatially constrain aggregation and promote organized spheroid formation and maintenance (Figure 4A). As expected, control DPSCs failed to form 3D spheroid on the micropatterned surfaces and instead developed multilayered two-dimensional cell sheets (Appendix Figure S3). In contrast, neuro-DPSCs formed uniformly positioned spheroids within the micropatterned geometry within 2 days post-seeding (Appendix Figure S4). Brightfield and immunofluorescence imaging demonstrated spheroid placement as designed and the formation of interconnected networks between neighboring spheroids. Neural-like DPSC spheroids extended aligned, neurite-like bundles, forming structured connections with neighboring spheres (Figure 4B, C). Both single bipolar and multi-bipolar neurite-like extensions were observed within the connecting tracts.

**Figure 4.**
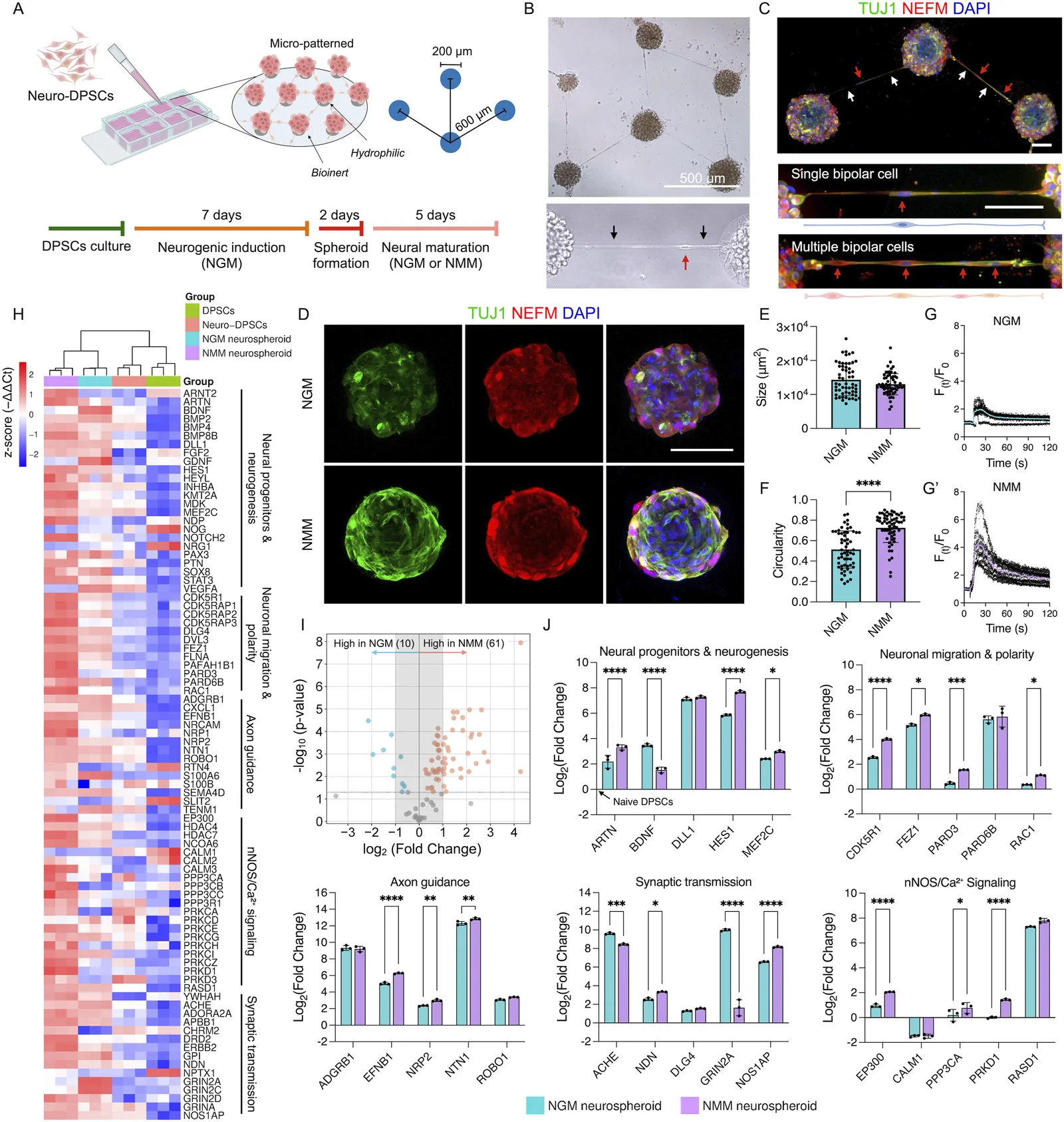
Micropatterned culture enables organized neural-like DPSC spheroid assembly, guided connectivity, and maturation-dependent transcriptional profile. (A) Schematic of the micropatterned culture workflow. (B) Brightfield and (C) tile-scanned, z-stack, maximum projection immunofluorescence images (Day 9) showing coordinated spheroid positioning at defined nodes and formation of neurite-like inter-spheroid connections. Higher magnification panels illustrate single bipolar and multi-bipolar neurite-like structures within the connecting tracts. (D) Cross-sectional immunofluorescent images demonstrating media-dependent spheroid morphology and neural marker expression patterns. (E, F) Quantification of spheroid size and circularity after NGM and NMM spheroid maintenance. Statistical analysis was performed using Welch’s t-test (n. of spheroid measured = 57 for NGM, 60 for NMM). (G, G’) Fluo-4-based calcium imaging traces showing changes in intracellular calcium levels in response to KCl stimulation (arrow). Each dot represents single spheroid (n = 10) while the lines indicate average values. (H) Heatmap of RT-qPCR array with unsupervised hierarchical clustering (Euclidean distance), comparing DPSCs, neuro-DPSCs, and neuro-like DPSC spheroids maintained in NGM or NMM. (I) Volcano plot demonstrating enhanced neural marker expression in spheroids matured in NMM compared to NGM. (J) Representative genes across functional modules (neural progenitors & neurogenesis, neuronal migration & polarity, axon guidance, synaptic transmission, and nNOS/Ca^2+^ signaling). Statistical analysis was performed by two-way ANOVA with Šídák’s multiple-comparisons test (n = 3). *p < 0.05, **p < 0.01, ***p < 0.001, ****p < 0.0001. Scale bar = 100 μm unless otherwise specified.

Following additional 5 days of maintenance in NGM and NMM conditions, the neuronal spheroids displayed media-dependent characteristics. Under NGM, neural-like spheroids showed a less compact, more heterogeneous architecture with an uneven ruffled boundary (Figure 4D). In contrast, NMM promoted the formation of more rounded and compact spheroids with a smoother, well-defined perimeter. Quantitative morphometric analysis showed that, although the mean spheroid size did not differ significantly between the two conditions, individual measurements under NMM were more tightly clustered around the mean and showed a smaller S.D., suggesting improved size uniformity (Figure 4E). Consistently, spheroid circularity was significantly higher under NMM (p < 0.0001), confirming a more geometrically uniform architecture (Figure 4F). Functionally, KCl-induced membrane depolarization triggered intracellular calcium responses in neural-like DPSC spheroids maintained in both NGM and NMM. However, calcium-imaging traces showed that NMM spheroids exhibited a markedly stronger depolarization-induced calcium transient, characterized by a rapid increase in fluorescence followed by gradual recovery (Figure 4G, G’). Quantitatively, the peak response in the NMM trace was approximately 3–4-fold higher than that observed in the NGM group, both of which were notably higher than 2D neuro-DPSCs. These findings suggest that NMM supports a more functionally responsive phenotype in neural-like DPSC spheroids, as reflected by enhanced KCl-induced calcium mobilization.

Gene expression profiling further supported neural-like signatures within patterned spheroids. Unsupervised clustering separated 2D control DPSCs and neuro-DPSCs from 3D neural-like spheroids and highlighted distinct transcriptional changes associated with neural progenitor regulation and neurogenesis, neuronal migration and polarity, axon guidance, synaptic transmission, and nNOS/Ca^2+^ signaling (Figure 4H). However, principal component analysis (PCA) as well as sample-to-sample correlation matrix indicated that NGM spheroids were transcriptionally more close to 2D neuro-DPSCs than NMM spheroids (Appendix Figure S5). Differential expression analysis indicated media-dependent modulation with NMM spheroids showing overall enhanced neuronal gene expression compared to NGM spheroids (Figure 4I). It confirmed upregulation of representative markers related to neural progenitors/neurogenesis, neuronal migration/polarity, axon guidance, synaptic transmission, and nNOS/Ca^2+^ signaling in both NGM and NMM spheroids compared to control DPSCs, with condition-specific differences between NGM and NMM (Figure 4J).

### 3.4. NMM spheroids upregulate synaptic machinery and neurotransmitter receptor gene networks

Based on the observation, NMM was selected for further characterization and functional gene network analysis. To assess whether neural-like DPSC spheroids in the micropatterned system exhibit neural functional indications, we analyzed expression of genes related to synaptic transmission machinery and neurotransmitter. Genes encoding key components of the presynaptic release and synaptic transmission machinery were broadly upregulated in NMM spheroids, including synaptic vesicle and active zone–associated factors (e.g., *SNPH*, *SV2A*, *SYN1*, *SYT*1, *BSN*, *CPLX1*, and *RIMS* family members) as well as SNARE-related genes (e.g., *STX1A*, *STX1B*, *STX3*, and *STXBP1*) (Figure 5A). Consistent with neurotransmitter pathway activation, genes involved in neurotransmitter synthesis and breakdown were also significantly increased in NMM spheroids (e.g., *ACHE*, *GAD1*, *MAOA*, *COMT*, and *TPH1*) (Figure 5B).

**Figure 5.**
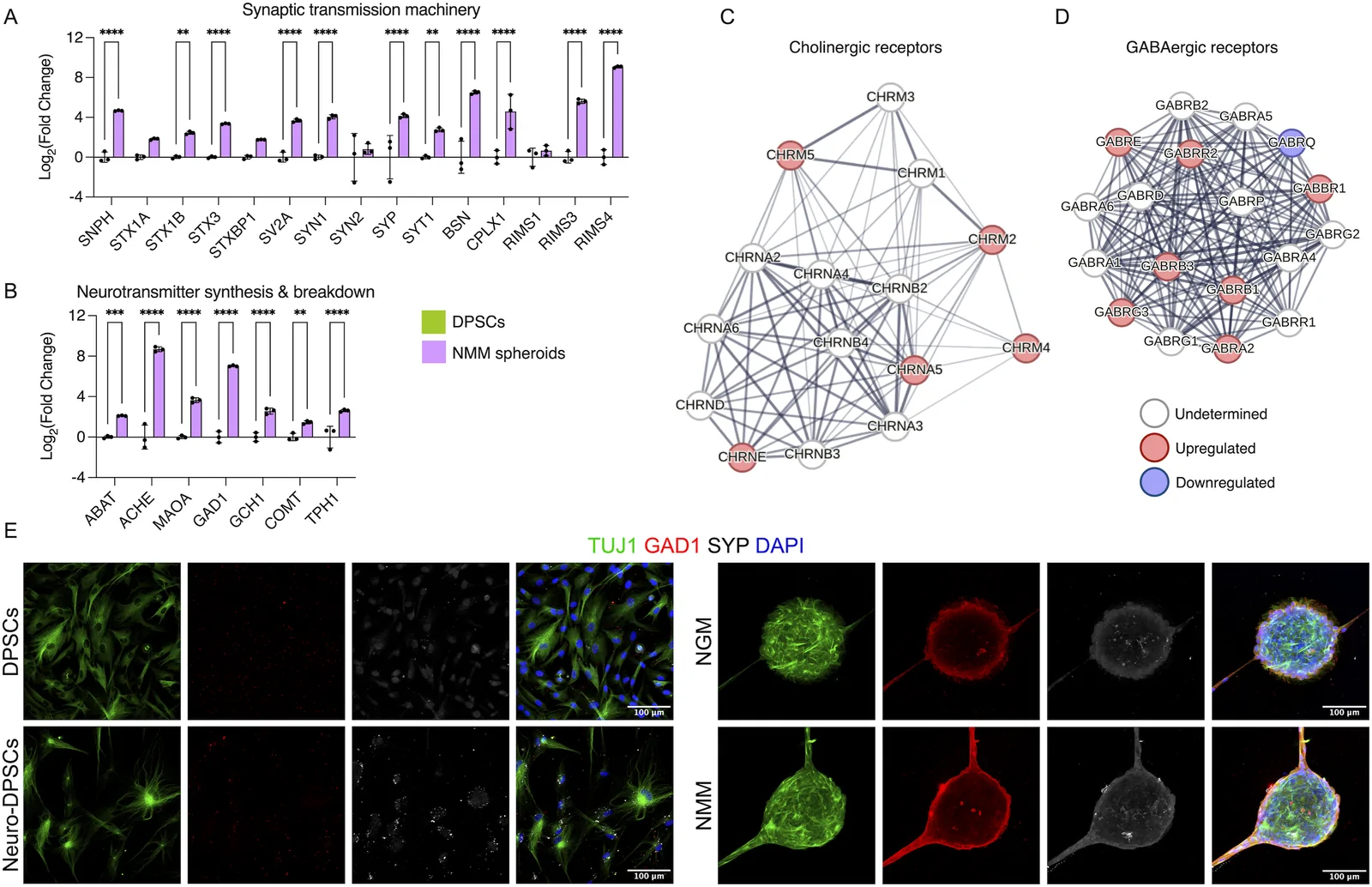
Neuro-like DPSC spheroid maintenance in NMM activates synaptic transmission and neurotransmitter signaling gene networks. (A, B) RT-qPCR analysis of genes encoding synaptic transmission machinery and (B) genes involved in neurotransmitter synthesis and breakdown in NMM spheroids compared with naïve DPSCs. (C, D) Receptor network maps illustrating coordinated regulation of cholinergic and GABAergic receptor gene families. Nodes represent receptor subunits and are colored according to expression change in NMM spheroids relative to DPSCs. (E) Immunofluorescence staining for GAD1, SYP, and TUJ1 in monolayer cultures of DPSCs and neuro-DPSCs, as well as in NGM and NMM spheroids. Statistical analysis was performed by two-way ANOVA with Šídák’s multiple-comparisons test (n = 3). *p < 0.05, **p < 0.01, ***p < 0.001, ****p < 0.0001.

Network analysis further demonstrated coordinated remodeling of neurotransmitter receptor gene families. In the cholinergic receptor network, multiple muscarinic (CHRM) and nicotinic (CHRN) receptor subunits were regulated, with several nodes (*CHRM2*, *CHRM4*, *CHRM5*, *CHRNA5*, *CHRNE*) upregulated in NMM spheroids (Figure 5C). More notably, the GABAergic receptor network showed widespread upregulation across GABR family members, indicating broad engagement of inhibitory neurotransmission-related receptor programs after spheroid maturation in NMM (Figure 5D).

Immunofluorescence coupled with the RT-qPCR array further supported the induction of GABAergic and synaptic markers in NMM spheroids. *GAD1*, which encodes a key enzyme responsible for GABA synthesis, showed a significantly lower Ct value in NMM spheroids (28.15 ± 0.20) than in DPSCs (34.91 ± 0.66; p < 0.0001), corresponding to a log_2_ fold change of 7.03 ± 0.07. Accordingly, GAD1 staining was prominent at the spheroid periphery and along neurite-like projections, whereas it was sparse in monolayered DPSCs and neuro-DPSCs. SYP staining was likewise enhanced in NMM spheroids (Figure 5E). Together, these findings suggest the acquisition of GABAergic and presynaptic-like molecular features in NMM spheroids.

## 4. Discussion

This study establishes a stepwise workflow that integrates neurogenic priming of DPSCs with micropattern-guided 3D assembly to generate spatially organized, depolarization-responsive neural-like spheroids. The novelty of this work does not lie in DPSC neurogenic induction, spheroid culture, or micropatterned cell culture alone, as each has been previously explored. Rather, the conceptual advance lies in combining these elements into a controlled workflow that links 2D neurogenic induction, micropattern-assisted self-assembly spheroid formation, guided inter-spheroid organization, maturation, and functional calcium-response analysis within a single platform. To our knowledge, this is the first report of DPSC-derived neural-like spheroids forming spatially defined neurite-like connections between neighboring spheroids.

DPSCs are an attractive adult stem cell source for neuro-regenerative modeling, although the extent and stability of true neural differentiation remain debated across protocols and readouts.^29,30^ Our data add to this field by showing that a 7-day 2D induction produces consistent neural-like morphological remodeling, increased expression of neuronal lineage markers, and activation of developmental signaling and neurotrophic programs. Morphological neurite-like features have been widely described in DPSC neuro-induction studies, but the literature also cautions that some induction cocktails can trigger cytoskeletal contraction or stress responses that mimic neuronal morphology without stable neuronal identity.^14,29,31,32^ By combining morphology with immunophenotyping, high-throughput gene expression, and excitability assessment, this study provides a comprehensive characterization of the previously underdocumented neural state of neuro-DPSCs beyond morphology alone. Our transcriptional results highlight activation of morphogen/developmental and progenitor-regulatory pathways, including components of Notch signaling (e.g., DLL1/HES/HEY).^33^ Notch dynamics are central to balancing neural progenitor maintenance versus differentiation in many neurodevelopmental contexts, with Hes/Hey transcriptional repressors acting downstream of Notch and interacting with Delta-like ligands (DLLs) in oscillatory or sustained regimes.^34–36^ The presence and modulation of these genes in neuro-DPSCs are consistent with a previously reported phenotype transition rather than immediate terminal differentiation.^36,37^ This interpretation aligns with prior reports that DPSCs can adopt neural progenitor-like features and secrete neurotrophic factors (e.g., BDNF, GDNF, and NGF), potentially supporting neural repair via paracrine actions even when full neuronal cell conversion is not achieved.^38^

The study also demonstrates a technical advance that initial neurogenic priming is essential for 3D self-organization, and that surface properties dictate whether cultures form adherent outgrowth assemblies (hydrophilic) or compact floating spheroids (hydrophobic). This behavior is consistent with general principles of cell aggregation and adhesion, where reduced substrate adhesion promotes cell–cell cohesion and spheroid formation, whereas adhesive surfaces support spreading and migration.^39,40^ In neural tissue engineering, engineered microenvironments are widely used to promote network formation and guide neurite extension through geometric constraints and patterned cues.^41,42^ Our data extend these concepts to adult stem cells, specifically DPSC-derived neural-like spheroids by showing that uncontrolled free-floating spheroids limit their maintenance and downstream analysis on hydrophobic surfaces, while micropatterning enables spatially fixed aggregates and reproducible inter-spheroid connectivity.

The micropatterned platform provides spatial control over spheroid positioning and inter-spheroid spacing, which are difficult to achieve in conventional free-floating aggregation systems.^43–45^ This challenge is particularly relevant for MSC-derived neural models, as protocols for neural induction and neural sphere/spheroid formation vary widely across the literature, and the reproducible generation of homogeneous neural-like populations remains unsolved.^46–49^ Beyond cellular heterogeneity, stochastic aggregation inherently limits experimental standardization because spheroids form at random locations, undergo uncontrolled fusion or fragmentation, and generate variable neurite outgrowth trajectories.^50^ In contrast, patterned neuronal culture systems and microstructured platforms are well-established bioengineering strategies to spatially constrain cell positioning, impose defined polarity, and guide neurite extension along predetermined paths, thereby improving structural control and reproducibility of *in vitro* neural circuits relative to unpatterned or randomly aggregated cultures.^43,51^ Seminal microcontact-printing work demonstrated that controlling adhesive nodes and non-adhesive regions can direct cell attachment and neurite routing, enabling engineered network topologies with increased predictability of connectivity.^52,53^ Additionally, pattern compliance and long-term stability are crucial as it enables reproducible neuronal network assays, including applications in toxicology testing and phenotypic screening, where variability in circuit geometry can otherwise confound functional readouts.^54,55^ In the platform we used, spheroids formed at UV-treated micropatterned surfaces as the points of contact and extended neurite-like tracts consisted of bipolar cells over bioinert non-adherent surfaces. This may particularly be advantageous to overcome the common issue of variance and reproducibility in spheroid size, spacing, and interconnectivity.

Beyond demonstrating micropattern-guided spheroid formation, this approach provides a spatially defined 3D model for investigating how geometric confinement, substrate properties, and soluble cues regulate DPSC self-organization, neurite-like tract formation, and neural-associated transcriptional programs.^56^ Its modular design could facilitate systematic screening of biomaterials, pattern geometries, and pro-neural conditions, thereby bridging conventional two-dimensional assays and more complex organoid or MSC-derived in vitro models.^57,58^ From a translational perspective, the ability to position and maintain spheroids in predefined architectures may support the development of standardized DPSC-derived constructs for neural-like and oral tissue engineering.^59–61^ However, progression from an experimental platform to a clinically deployable therapeutic product will require scalable production using chemically defined, xeno-free, and good manufacturing practice-compliant processes, together with validated critical quality attributes and mechanism-relevant potency and batch-release assays.^9^ These requirements are particularly important because the clinical evidence for dental tissue-derived cell products remains concentrated in dental pulp, periodontal, and craniofacial regeneration rather than neurological indications.^9,62^ Ultimately, regulatory classification, manufacturing complexity, cost-effectiveness, and reproducibility across centers will determine whether such constructs can advance beyond proof-of-concept studies.

Several limitations should be acknowledged. First, this study primarily relied on morphological assessment, neuronal-associated marker expression, and transcriptional profiling, while direct functional evaluation was limited to KCl-induced depolarization-responsive calcium signaling. Accordingly, the present protocol is not intended to demonstrate acquisition of bona fide functional neuronal identity or mature neuronal electrophysiological properties. Importantly, a KCl-evoked increase in intracellular Ca^2+^ demonstrates membrane depolarization-associated calcium entry and handling but is not equivalent to neuronal excitability, which requires direct evidence of voltage-gated ionic currents and action-potential firing. Indeed, KCl-responsive calcium signals have also been observed modestly in undifferentiated DPSCs, indicating that this response is not specific to functionally differentiated neurons.^63,64^ In contrast, Pardo-Rodríguez et al. applied retinoic acid and repeated KCl pulses during prolonged differentiation for up to 60 days and demonstrated TTX-sensitive Na^+^ currents, voltage-dependent K^+^ currents, spontaneous electrical activity, and repetitive action potentials with recovery to the baseline membrane potential using whole-cell patch-clamp analysis.^31^ These electrophysiological endpoints were not assessed in the present study. Therefore, the calcium responses observed here should not be interpreted as evidence of action-potential competence or mature neuronal excitability. Moreover, neither KCl-responsive calcium signaling nor the expression of synaptic markers demonstrates functional synaptic connectivity, which would require direct detection of synaptic currents and coordinated network activity by patch-clamp or microelectrode array (MEA) analysis. Notably, the integration of DPSC-derived neuronal-like cells into functional synaptic networks also remains unresolved.^31^ It is noteworthy that, the micropatterned platform used in the study is currently not compatible with existing electrophysiological analytic systems (i.e., MEA). However, integrating micropatterned surfaces into tailored MEA systems for both 2D and 3D assays have been proposed and successfully developed.^65–67^ In our approach, the spheroids have physical contact with the substrate at the points of contact, possibly allowing the electrophysiological measurement through integrated electrodes, which is not feasible in free-floating spheroids or with limited adhesion sites (i.e. U-bottom/ultra-low attachment plates). Compared with iPSC-derived neuronal organoids, which are designed to recapitulate aspects of CNS development and can exhibit greater neural-cell diversity, action potentials, synaptic currents, and coordinated network activity following prolonged culture,^68–70^ the present DPSC spheroid system was not intended to model mature neural tissue or generate fully mature neurons. Rather, it was developed as a bioengineering platform to facilitate the reproducible generation, spatial organization, and maintenance of DPSC-derived spheroids.^57^

Second, DPSC populations are heterogeneous and donor-dependent, and therefore bulk readouts can mask subpopulations or alternative lineage trajectories. Our readout through calcium imaging exhibited the subpopulation of neuro-DPSCs with high excitability. Prior work comparing DPSC neurogenic differentiation also emphasized variability and the need for rigorous benchmarking.^9,29^ Furthermore, although this study used pooled-donor DPSCs to average donor-to-donor variability, donor-specific phenotypes should be further examined in future translational studies. The DPSCs used in the study were derived from premolars of juvenile female donors with completed root formation. Recent multi-omics evidence suggests that sex may contribute to epigenomic and transcriptomic variation in DPSCs.^71^ Other factors, including donor age, oral and systemic health, and tooth developmental stage, may also influence cellular responses to the biofabrication approach. Therefore, the present findings may not be directly generalizable to DPSCs isolated from other donor populations or from teeth at different developmental stages, such as immature third molars.^9,22^ Lastly, while micropatterning improves organization, it also imposes geometric constraints that may bias neurite alignment and gene expression. Future studies should systematically compare alternative pattern geometries, dimensions, and inter-spheroid spacing, together with matched non-patterned controls, to identify configurations that best support spheroid uniformity, connectivity, and long-term maintenance.

## 5. Conclusion

In this study, we established a strategy to develop DPSCs into spatially organized, depolarization-responsive spheroids. Neurogenic induction induced pronounced morphological remodeling and a shift towards neural-like lineage marker expression and neurodevelopmental transcriptional program. Neuro-DPSCs showed an enhanced capacity for self-assembly of 3D spheroids compared with control DPSCs, but conventional hydrophobic suspension conditions produced free-floating spheroids that limited long-term culture. To address this limitation, we implemented a micropatterned chip that spatially constrained aggregation, enabled uniform spheroid placement, and promoted guided neurite-like interconnections. Importantly, spheroid maintenance medium strongly influenced spheroid organization, with our tailored maintenance cocktail, NMM, supporting more compact and structurally organized neural-like spheroids with enhanced neural marker expression and depolarization-responsive calcium activity. However, further electrophysiological and synaptic functional validation will be required to determine whether these molecular features correspond to mature neuronal network activity.

Collectively, our findings demonstrate that combining neurogenic priming with micropattern-assisted assembly provides a practical route to reduce variability in neural-like spheroid cultures and enables long-term maturation within a controlled circuit geometry. This platform offers a scalable foundation for future functional validation and for applications requiring standardized 3D neural constructs, including mechanistic studies, medium optimization, and high-content screening.

## Supplemental material

Supplemental material - Generation of depolarization-responsive dental stem cell-derived neural-like spheroids using a micropatterned chip platform Supplemental material for Generation of depolarization-responsive dental stem cell-derived neural-like spheroids using a micropatterned chip platform by Normann Misje. Roman, Åshild Johansen, Niyaz Al-Sharabi, Inge Fristad, Hiroshi Egusa, Ana Angelova Volponi, Shuntaro Yamada in Journal of Tissue Engineering

Supplemental material - Generation of depolarization-responsive dental stem cell-derived neural-like spheroids using a micropatterned chip platform Supplemental material for Generation of depolarization-responsive dental stem cell-derived neural-like spheroids using a micropatterned chip platform by Normann Misje Roman, Åshild Johansen, Niyaz Al-Sharabi, Inge Fristad, Hiroshi Egusa, Ana Angelova Volponi, Shuntaro Yamada in Journal of Tissue Engineering

Supplemental material - Generation of depolarization-responsive dental stem cell-derived neural-like spheroids using a micropatterned chip platform Supplemental material for Generation of depolarization-responsive dental stem cell-derived neural-like spheroids using a micropatterned chip platform by Normann Misje Roman, Åshild Johansen, Niyaz Al-Sharabi, Inge Fristad, Hiroshi Egusa, Ana Angelova Volponi, Shuntaro Yamada in Journal of Tissue Engineering

Supplemental material - Generation of depolarization-responsive dental stem cell-derived neural-like spheroids using a micropatterned chip platform Supplemental material for Generation of depolarization-responsive dental stem cell-derived neural-like spheroids using a micropatterned chip platform by Normann Misje Roman, Åshild Johansen, Niyaz Al-Sharabi, Inge Fristad, Hiroshi Egusa, Ana Angelova Volponi, Shuntaro Yamada in Journal of Tissue Engineering
