# Supplementary material for: Generation of depolarization-responsive dental stem cell-derived neural-like spheroids using a micropatterned chip platform
Source: J Tissue Eng. 2026 Oct 7;17:20417314261494165. doi: 10.1177/20417314261494165 (PMC13770044; doi:10.1177/20417314261494165)
Supplement: Supplementary file 1 [file sj-pdf-1-tej-10.1177_20417314261494165.pdf]

Generation of depolarization-responsive dental stem cell-derived neural-like spheroids using a micropatterned chip platform

Normann Misje Roman[1], Åshild Johansen[1], Niyaz Al-Sharabi[1], Inge Fristad[1], Hiroshi Egusa[2,3], Ana Angelova Volponi[4], Shuntaro Yamada[1,4*]

1. Center of Translational Oral Research, Department of Clinical Dentistry, University of Bergen, Bergen, Norway
2. Center for Advanced Stem Cell and Regenerative Research, Tohoku University Graduate School of Dentistry, Miyagi, Japan.
3. Division of Molecular & Regenerative Prosthodontics, Tohoku University Graduate School of Dentistry, Miyagi, Japan.
4. Centre for Craniofacial & Regenerative Biology, Faculty of Dentistry, Oral & Craniofacial Sciences, King's College London, London, UK

*Correspondence
Shuntaro Yamada: Shuntaro.yamada@uib.no
Center of Translational Oral Research, Department of Clinical Dentistry, University of Bergen, Årstadveien 19, 5009 Bergen, Norway

1. Appendix Materials and Methods

1.1. Transcriptomic characterization of pooled DPSCs relative to individual donors

To evaluate whether the pooled DPSC population used in the study represents general DPSC populations, RNA sequencing was performed and analyzed as previously reported [1], and the pooled sample was compared with DPSCs derived separately from 14 individual donors. Principal component analysis was conducted using the 5,000 genes with the highest variance across all samples.

2. Appendix Results

2.1. DPSC characterization

We validated the baseline transcriptomic profile of the pooled DPSCs against DPSCs from 14 individual donors. PCA showed no pronounced separation among individual donors along PC1 and PC2, while the pooled samples from the five donors clustered near the center of the contributing-donor distribution, supporting the pooled population as a representative average that minimizes donor-specific effects (Fig. S1A). In culture, the pooled DPSCs displayed a typical fibroblast-like, spindle-shaped morphology and tri-lineage differentiation capacity (Fig. S1B).

Flow cytometry confirmed that the DPSCs used in the study fulfilled the immunophenotypic criteria for MSCs [2]. DPSCs showed negligible expression of the hematopoietic markers CD34, CD45, and HLA-DR (< 5%) while exclusively expressed CD73, CD90, and CD105 (>95%) (Fig. S1C).

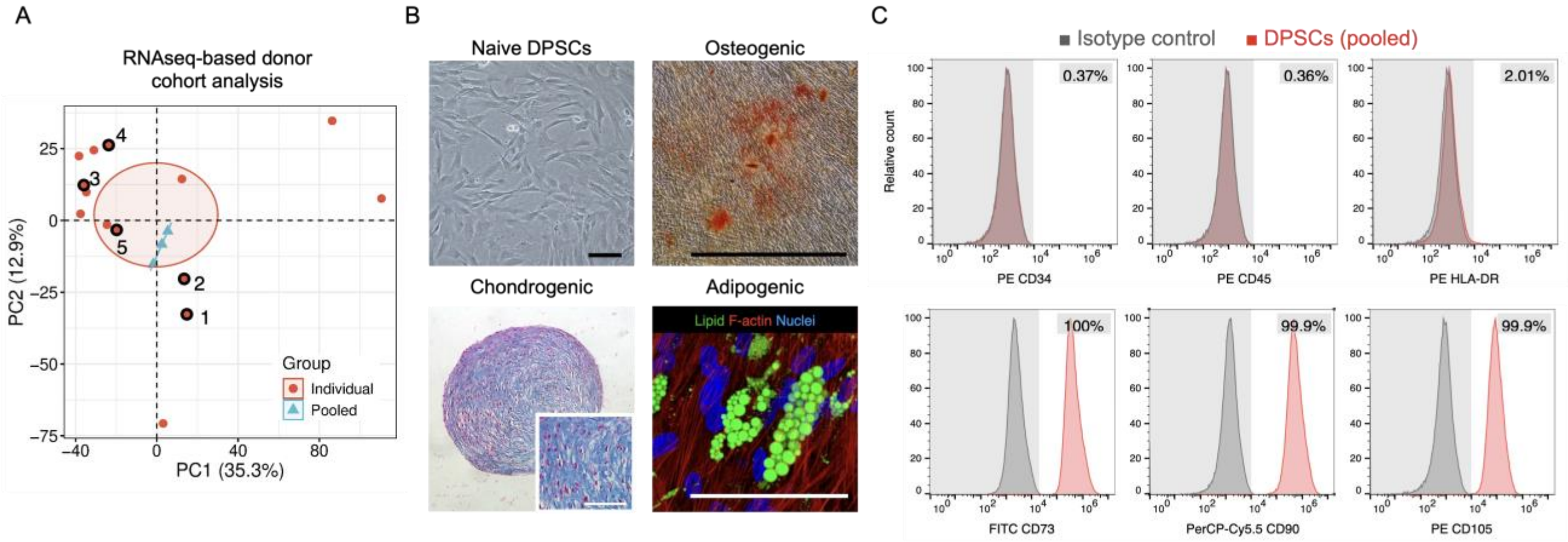

Figure S1. Characterization of pooled dental pulp stem cells (DPSCs) used in the study. (A) Principal component analysis of transcriptomic profiles from the pooled DPSC population used in the study and 14 individually processed DPSC donor samples, performed using the 5,000 genes with the highest variance across all samples. The five donors contributing to the pooled population are indicated by numbered points, and the pooled samples are shown as triangles. The ellipse represents the confidence ellipse for individual and pooled DPSCs. (B) Representative images of pooled, naïve DPSC morphology and tri-lineage differentiation confirmed by mineral deposition (Alizarin Red S staining), proteoglycan-rich chondrogenic pellets (Alcian Blue staining), and intracellular lipid droplet accumulation (Bodipy staining). Scale bars = 100 µm. (C) Immunophenotyping of pooled DPSCs by flow cytometry.

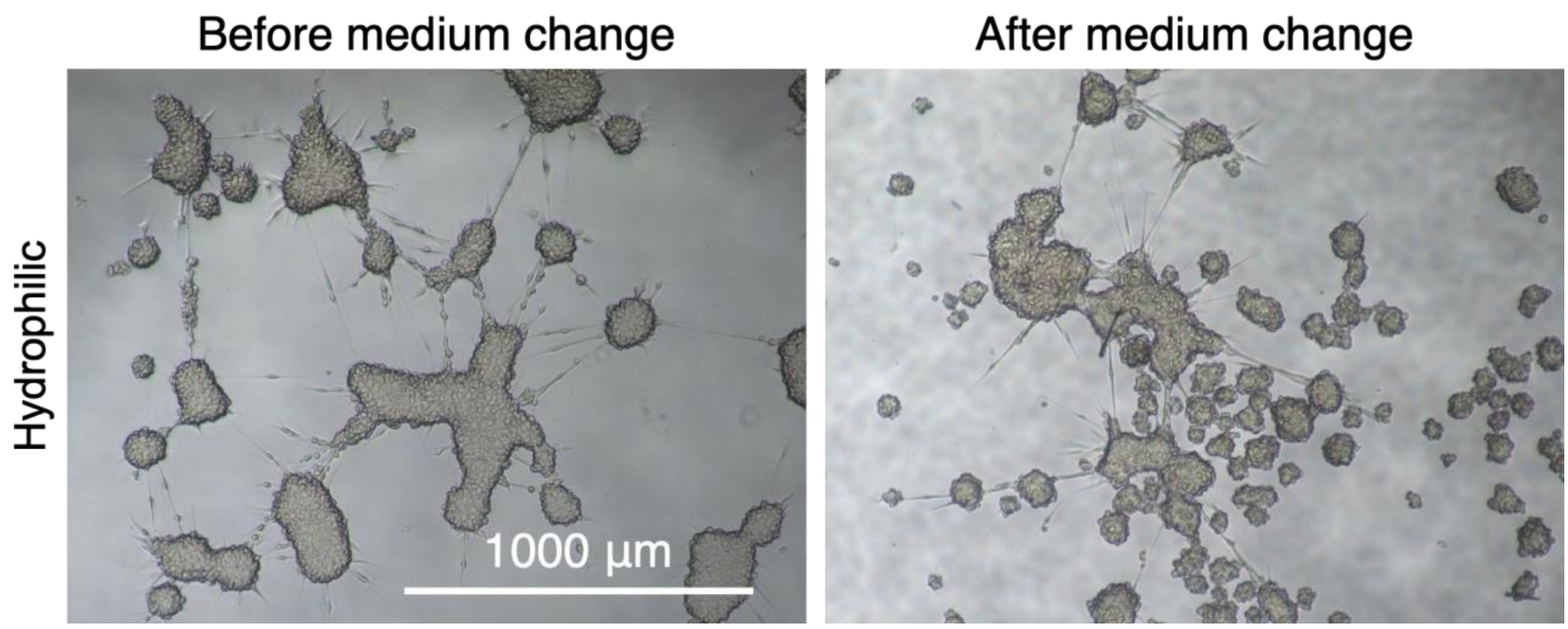

Figure S2. Bright-field images of self-assembled neural-like DPSC spheroids cultured on hydrophobic surfaces. After 48 h, neurogenic DPSCs formed interconnected spheroid-like assemblies linked by neurite-like cellular extensions. Because the spheroids remained non-adherent, fluid movement during medium exchange, particularly that generated by pipetting, rapidly disrupted the spatial organization of these assemblies

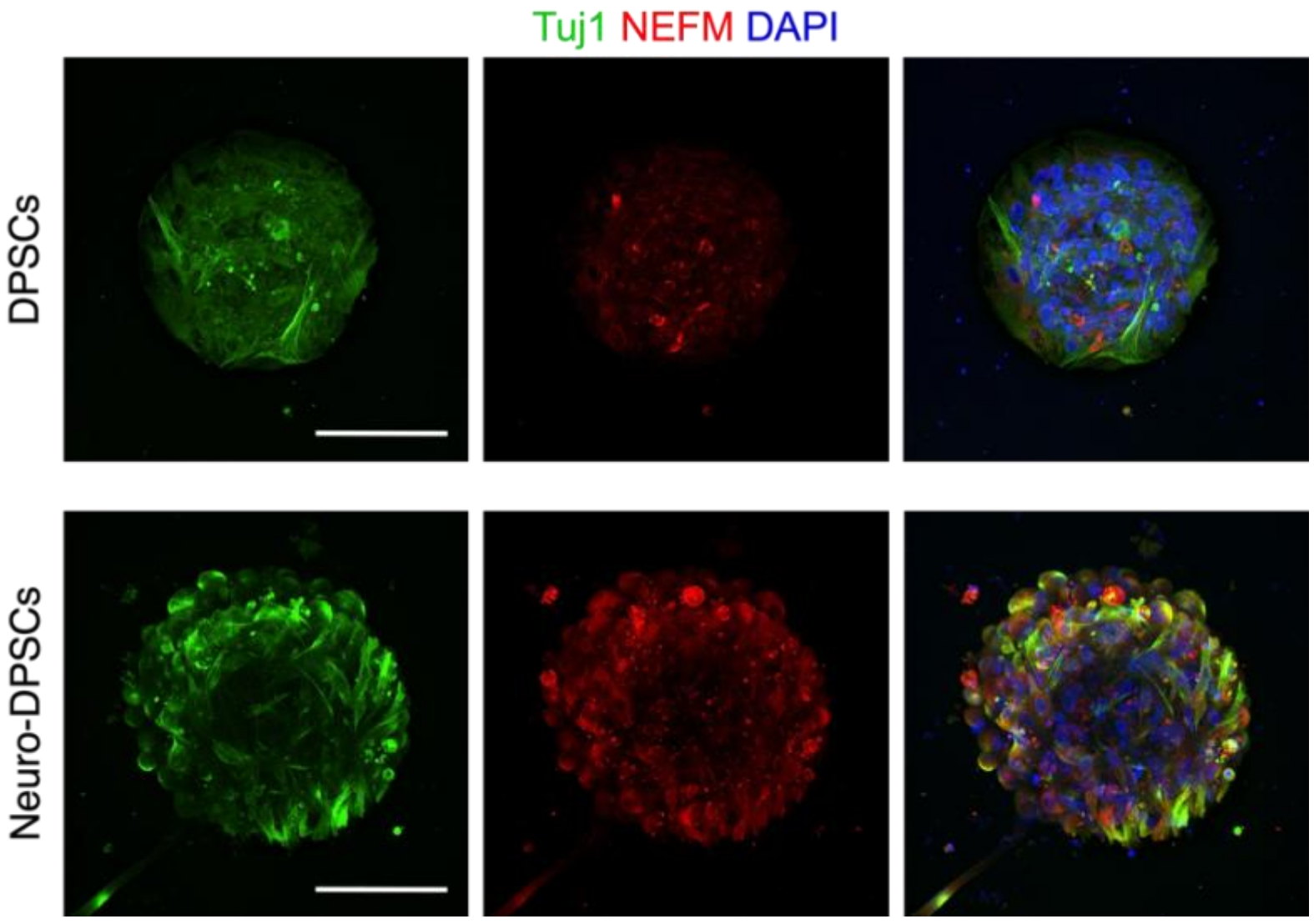

Figure S3. Microscopy and morphometric analysis of micropattern-assisted 3D assembly by DPSCs and neuro-DPSCs. Z-stack immunofluorescence images of cells cultured on micropatterned surfaces 2 days after seeding. Non-induced DPSCs showed flat multi-layered cell adhesion yet failing 3D spheroid assembly, whereas neuro-DPSCs formed compact spheres interconnected with polarized neuronal-like cells. Scale bar, 100 µm.

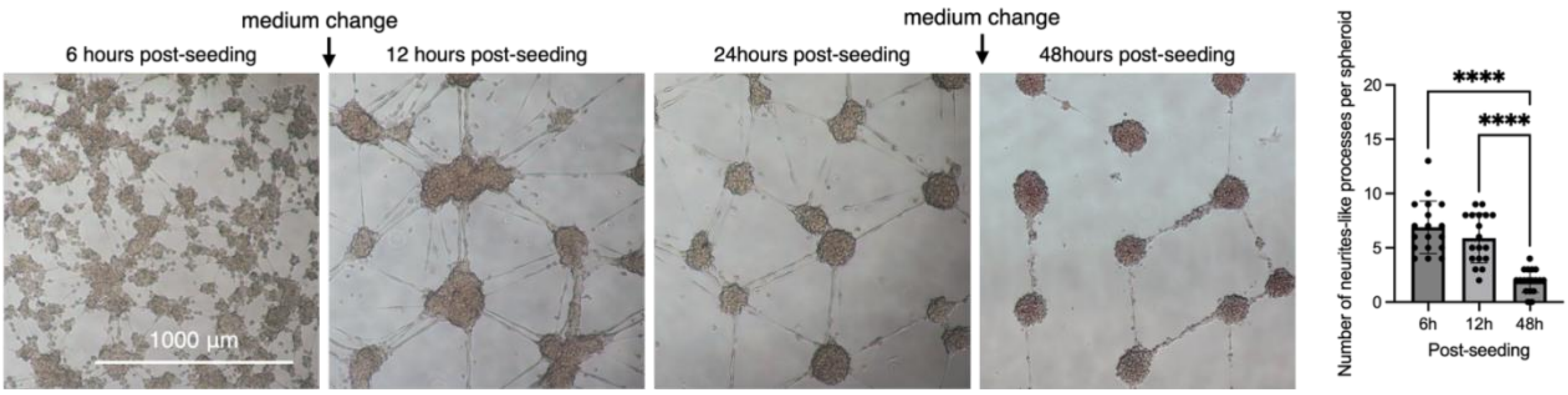

Figure S4. Self-assembly of neuro-DPSCs into neural-like spheroids on micropatterned surfaces. Representative bright-field images showing the progressive organization of initially dispersed neuro-DPSC aggregates into compact spheroids interconnected by elongated neurite-like cellular extensions. The micropatterned surface guided the spatial arrangement of the spheroids and promoted the formation of network-like structures. Due to non-repairable natures of the neurite-like structure, the number of interconnected spheroids decreased over time, particularly by stress caused by handling (e.g., medium change). Images are presented in chronological order from left to right. Eighteen spheroids from three independent chips were quantified by ImageJ/Fiji. Statistical significance was assessed using one-way ANOVA with Šídák's multiple comparisons test. ****$p < 0.0001$.

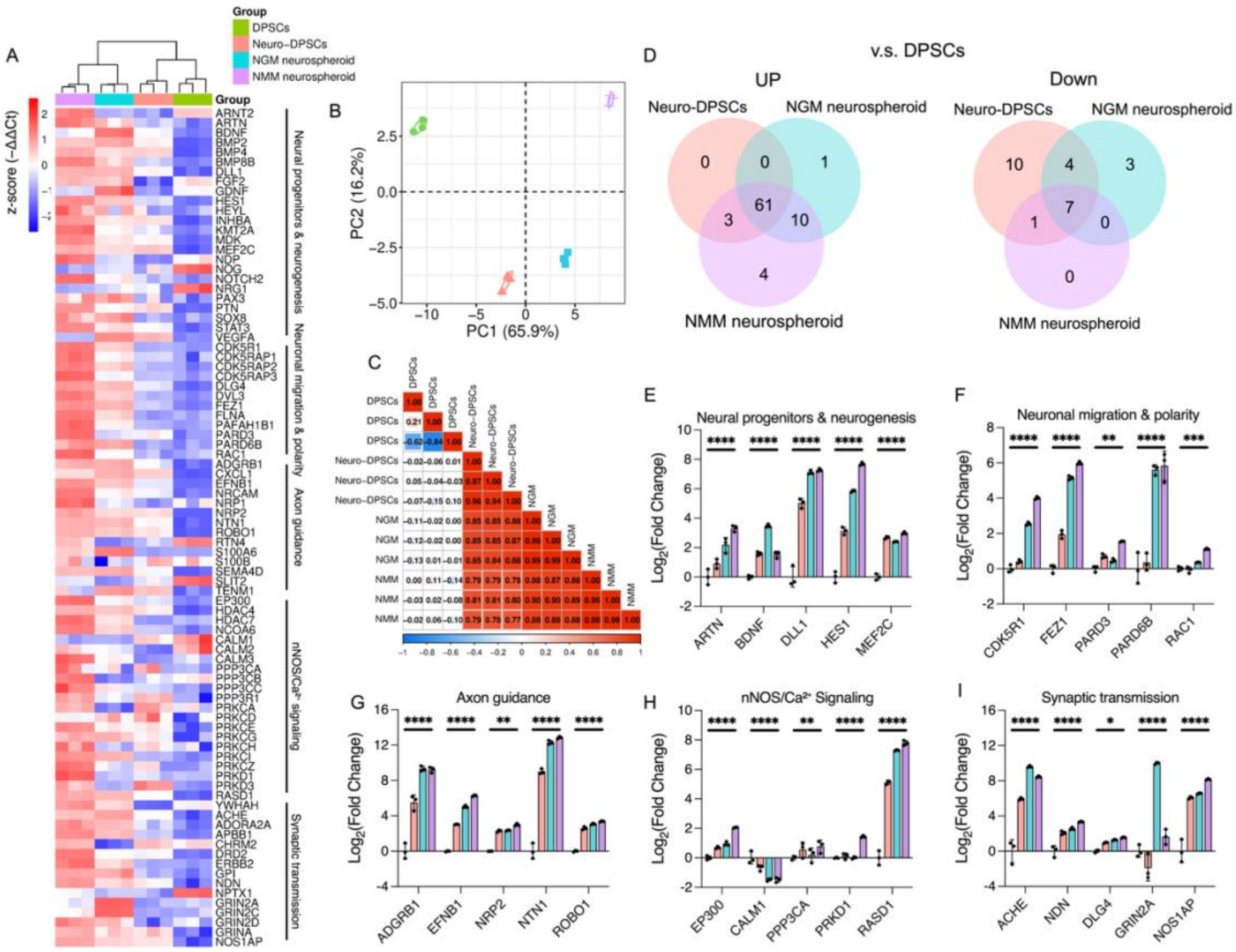

Figure S5. Multivariate and pathway-level RT-qPCR array across DPSCs, neuro-DPSCs, and neurospheroids. (A) Heatmap of RT-qPCR expression values with unsupervised hierarchical clustering (Euclidean distance). (B) Principal component analysis (PCA) of the RT-qPCR dataset showing separation of experimental groups along PC1 and PC2. (C) Sample-to-sample correlation matrix (Pearson correlation coefficients).(D) Venn diagrams summarizing the overlap of significantly upregulated (left) and downregulated (right) genes in neuro-DPSCs, NGM neurospheroids, and NMM neurospheroids compared with DPSCs. (E–I) Genes expression of functional modules: neural progenitors & neurogenesis, neuronal migration & polarity, axon guidance, nNOS/Ca^{2+} signaling, and synaptic transmission. *$p < 0.05$, **$p < 0.01$, ***$p < 0.001$, ****$p < 0.0001$.

Appendix reference

[1] S. Yamada, K. Holomková, N. Al-Sharabi, T. Qiu, H. Egusa, I. Fristad, A. Angelova Volponi, Sex as a biological variable in human dental pulp stem cells: An exploratory epigenomic and transcriptomic comparison, Regenerative Therapy 32 (2026) 101117.

[2] M. Dominici, K. Le Blanc, I. Mueller, I. Slaper-Cortenbach, F. Marini, D. Krause, R. Deans, A. Keating, D. Prockop, E. Horwitz, Minimal criteria for defining multipotent mesenchymal stromal cells. The International Society for Cellular Therapy position statement, Cytotherapy 8(4) (2006) 315-7.
